# Nano-Silica Sol-Gel and Carbon Nanotube Coupling Effect on the Performance of Cement-Based Materials

**DOI:** 10.3390/nano7070185

**Published:** 2017-07-14

**Authors:** Weiwen Li, Weiming Ji, Forood Torabian Isfahani, Yaocheng Wang, Gengying Li, Yi Liu, Feng Xing

**Affiliations:** 1 Guangdong Key Provincial Durability Center for Marine Structure, Shenzhen Durability Center for Civil Engineering, Department of Civil Engineering, Shenzhen University, Shenzhen 518060, China; liweiwen@szu.edu.cn (W.L.); liuyihnu86@gmail.com (Y.L.); xingf@szu.edu.cn (F.X.); 2 Department of Architecture and Civil Engineering, City University of Hong Kong, Kowloon 999077, Hong Kong, China; weimingji2-c@my.cityu.edu.hk; 3 Dipartimento di Chimica, Materiali e Ingegneria Chimica “G. Natta” via Mancinelli, Politecnico di Milano, 7-20131 Milano, Italy; forood.torabian@polimi.it; 4 College of Water Conservancy and Civil Engineering, South China Agricultural University, Guangzhou 510642, China; gyli@stu.edu.cn

**Keywords:** carbon nanotubes (CNTs), nano-silica (NS), interfacial adhesion, nucleation effect, energy dissipation

## Abstract

Carbon nanotubes (CNTs) have shown promise for improving the mechanical performance of cement composites through crack-bridging and frictional pull-out. The interactive behaviors between CNTs and cement matrix act are crucial in optimizing the reinforcement of CNTs in cement composites. This study investigates the effects of nano-silica (NS) sol-gel on the interactive behaviors of CNTs and the cement matrix through a series of experiments and analyses. UV-visible spectrometer results show that CNTs are well-dispersed in suspension and the addition of NS has a negligible effect on the stability of CNT dispersion. Calorimetry tests and dynamic mechanical analysis demonstrate the nucleation and frictional performance of CNTs in cement matrix, respectively. The paper shows that the physical adsorption of NS on the CNT surface could result in the acceleration of cement hydration. Morphology observation confirms that a denser interface between CNTs and cement hydrates is formed. Finally, the improved interaction between CNTs and cement hydrates leads to a substantial increase in friction between CNTs and the cement matrix under periodic loading. NS may act as an ideal admixture for improving both the interactive behaviors between CNTs and cement matrix and the damping properties of cement composite.

## 1. Introduction

Concrete is used as a structural material due to its low cost, wide availability and ideal mechanical properties. However, its most important ingredient, hydrated cement paste, is a quasi-brittle material and has disadvantages such as low tensile strength, low ductility and susceptibility to cracking due to early-age shrinkage, which would reduce the mechanical performance of concrete and the service life of concrete structures.

Adding fibers to cement-based materials to develop crack-free concretes with improved ductility is a strategy that has gained considerable attention worldwide in recent years. Among different fiber materials, carbon nanotubes (CNTs) are a promising candidate due to their excellent mechanical, thermal and electrical properties. Theoretically, for an individual CNT, the Young’s modulus and tensile strength could reach 1 TPa and 100 GPa, respectively, with an ultimate strain of up to 15% and specific surface area of up to 1000 m^2^/g [1,2,3]. To date, many investigations have shown the excellent mechanical and electrical properties of CNTs when they are added to cement-based composites [4,5,6,7,8,9,10].

However, the tendency that CNTs have to agglomerate is still a key issue when they are dispersed in a cement matrix due to their high specific surface area and van der Waals’ force. This poor dispersion confines CNTs as reinforcements in the hydrated cement paste. Various physical and chemical techniques have been introduced to assist with the dispersion of CNTs, such as ultra-sonication, ball-milling, surface functionalization, ionic surfactant and non-ionic surfactant methods [11,12,13,14,15,16,17]. The mechanisms for these dispersion methods could be explained as follows: ultra-sonication releases high levels of energy, which generate microscopic bubbles in suspension, and the bubbles separate individual CNTs from bundles [18]; ball-milling process would charge the surface of CNTs and enable the individual CNTs insert into the composite [19]; using functional groups introduced by surface functionalization and ionic surfactants could form micelles around the CNTs and inhibit its aggregation [20]; the non-ionic surfactants could be absorbed into the surface of CNTs, which contributes to exfoliating individual CNTs from bundles through a steric stabilization effect [14]. Aside from these mentioned methods, another optional method is dispersing CNTs in water with non-covalent functionalization surfactant, which has the characteristic of not changing the inherent electrical and mechanical properties of CNTs [15]. 

The performance of CNTs/cement composite is not only influenced by the dispersion of CNTs in cement paste, but also related to their interaction. Computational simulation analyses demonstrated that CNTs will provide good reinforcement when the matrix is under loading, when a good connection between the CNTs and matrix is formed [21]. Furthermore, Al–Rub et al. [22] used the finite element simulation method to investigate the key parameters that control the pull-out behavior of a single CNTs from cement matrix and found that the interfacial adhesion governs the pull-out strength. However, due to its smooth surface at an atomic scale [23], the CNTs shows little potential for forming a strong interface with a cement matrix. Few researchers have concentrated on the bonding strength between CNTs and the cement matrix in order to obtain ideal reinforcement of CNTs. The interfacial adhesion between CNTs and cement matrix may be improved either by functional treatment on the CNT surface or by adding additional materials in the composite. Previous efforts have been made to introduce hydroxyl, carboxyl and silica functional groups to the CNT surface for the purposes of higher interfacial strength between CNTs and cement matrix and greater mechanical enhancement of cement composites [24,25,26,27]. Also, due to small particle size and pozzolanic potential, silica fume is used to densify the interface between CNTs and cement matrix and enhance the capacity of load transfer and the overall strength of the cement composite [28]. However, it is reported that silica functional groups actually retard the nucleation effects of CNTs during cement hydration [27]. Although existing studies have shown the potential role of the silica-functional group in the interfacial bond between CNTs and the cement matrix, the probable role of NS in enhancement of interfacial bonds between CNTs and the cement matrix has not been widely studied in the current literature. As stated by Makar and Chan [29], the nucleation of hydration products, calcium silicate hydrate (C–S–H), during the acceleration period by the CNT bundles is responsible for the reinforcements to the matrix. Makar et al. [30] also prove that development of the strength of the matrix is due to the increase in the nucleation effect to the point where C–S–H is held together by the CNTs, producing a dense C–S–H structure against the surfaces of CNTs and leading to crack bridging. The influence of NS on the interaction between CNTs and the cement matrix are not well known, and efficient means for improving damping properties of CNTs/cement composite are urgently needed. Our study will close the knowledge gap concerning the improvement of interfacial interaction between CNTs and cement matrix and provide useful information to alleviate the dynamic vibration response and damage or fatigue in order to meet people’s safety and comfort needs.

In this work, the effects of NS on CNT performance, including nucleation and energy dissipation in the cement matrix, are studied systematically. Dispersion of CNTs in suspension is characterized with a UV-visible spectrophotometer. The influence of nano-silica (NS) on the microstructure and hydration promotion of CNT-reinforced cement pastes are investigated by means of scanning electron microscopy (SEM) and calorimetry tests, respectively. Dynamic mechanical analysis (DMA) is utilized to study the effects of NS on the energy dissipation behavior of CNTs in the cement matrix. The experimental program, analyses and discussions on the results are introduced in detail in this paper as well. 

## 2. Results

### 2.1. Characterization of Multi-Walled CNT (MWCNT) Dispersion in Aqueous Suspension

**Figure 1:**
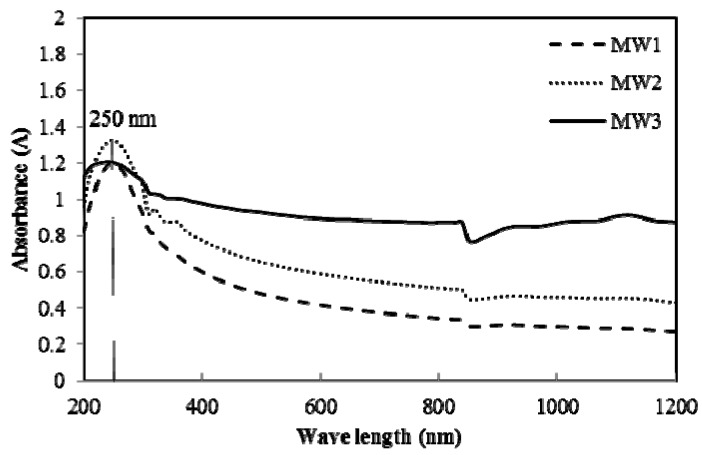
Ultraviolet–Visible (UV-Visible) absorbance of multi-walled carbon nanotubes (MWCNTs) suspension (MW1 refers to 0.02% MWCNTs without nano-silica (NS); MW2 refers to 0.02% MWCNTs with NS; MW3 refers to 0.08% MWCNTs with NS).

In this study, the definitions of samples are explained in Table 1. Figure 1 presents the Ultraviolet-Visible (UV-Visible) Spectroscopy results for MWCNT aqueous suspensions. The characteristic absorbance peak is an indicator for concentration of individual MWCNTs obtained in the suspension. As seen in Figure 1, the characteristic peak (at a wave length of 250 nm) of MW3 (prepared using NS gel) had close intensity as MW1 which was pure MWCNTs suspension. For both of the samples adding NS, an increase in the concentration of MWCNTs from 0.02% (the MW2 samples) to 0.08% (the MW3 samples) led to a slight decrease in absorbance. Table 2 shows the intensity of the characteristic peak at the wave length of 250 nm and its change during the initial 48 h standing. It can be observed that the intensity of the characteristic peak, which compares the concentration of individually dispersed MWCNTs, kept consistent along with the standing duration when the dosage of MWCNTs is 0.02% by weight of cement. However, MWCNTs with a dosage of 0.08% by weight of cement decreased in the intensity as time went by.

The results indicate that addition of NS can negligibly affect the dispersion and stability of MWCNTs in aqueous suspension. For the MWCNT dispersion in NS gel, the optimal dosage of MWCNTs in this study is 0.02% by weight of cement. At a higher MWCNT content, its stability is poor and the NS seems to have slight negative effect on the dispersion in the long term. With a higher MWCNT content, the distance between dispersed individual MWCNTs might diminish, which may lead to a stronger Van del Waals’ force and hence to easier re-agglomeration of MWCNTs.

### 2.2. Heat of Hydration Test

**Figure 2:**
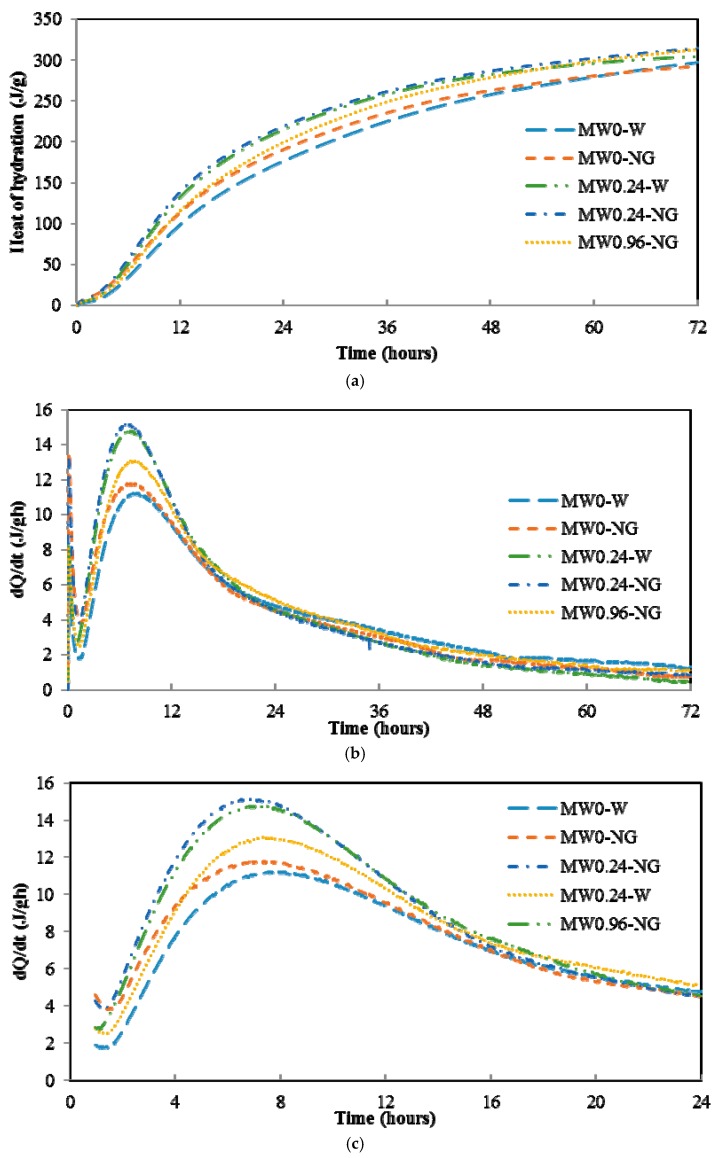
Generation of heat during initial hydration. (**a**) Cumulative heat curves for 72 h hydration; (**b**) Rate of hydration; (**c**) Heat flow during 1–24 h.

In this study, the definitions of samples are explained in Table 3. Figure 2 presents the kinetics of the cement hydration of various samples during the initial 72 h, and the total amounts of heat released are listed in Table 4. As seen from Table 4 and Figure 2a, the total amount of heat released during the initial 72 h of hydration follows a descending order of MW0.24-NG (0.02% MWCNT + NS) ≈ MW0.24-W (0.02% MWCNT) > MW0.96-NG (0.08% MWCNT + NS) > MW0-W > MW0-NG (NS). The similar results of MW0.24-NG and MW0.24-W further confirm the dispersion characterization above. For the three samples containing MWCNTs, the less-dispersed MW0.96-NG (using MW3 suspension) seems to slightly decelerate the hydration of cement at the early stage (Figure 4a). The agglomerated CNTs will exhibit smaller specific surface areas and provides fewer precipitation sites for hydration products.

The acceleration period during the first day of calorimetry curves can be assumed to be mainly the hydration of C_3_S [29]. As seen in Figure 2c, it was found that the extent of C_3_S hydration followed a descending order of MW0.24-NG > MW0.96-NG > MW0.24-W > MW0-NG > MW0-W. The hydration reaction can be accelerated both by chemical means such as chemical reaction or through the addition of nucleating agents, which promotes the formation of hydration products by offering more sites for the reaction. It is clear from the data in Figure 2c that incorporation of MWCNTs promoted hydration of cement in the composite, which is consistent with other reported findings [11,29]. MWCNTs are physically inert and do not participate in the hydration process; they were assumed to act as nucleation spots for formation of C–S–H, and the C–S–H appears to have formed preferentially around the MWCNTs, which accelerates the formation rate of the C–S–H. Furthermore, a distinct increase in C–S–H formation is detected in samples of MWCNTs dispersed by NS. It should be noted that addition of NS only marginally enhances C–S–H formation.

### 2.3. Morphology Observation

**Figure 3:**
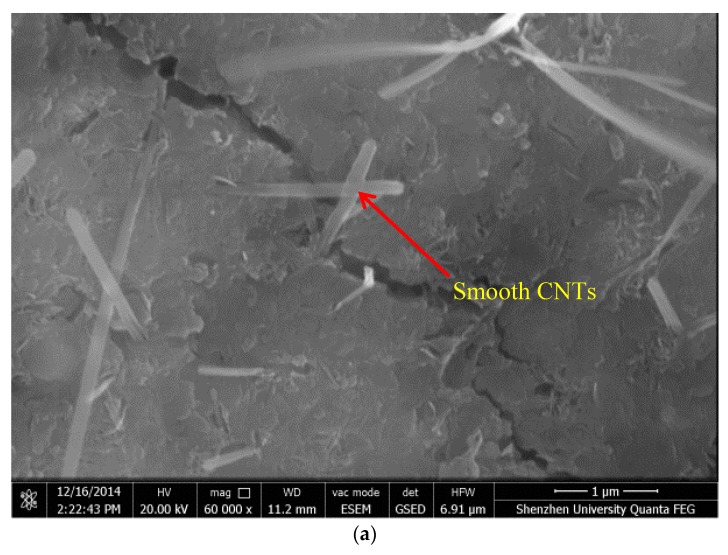

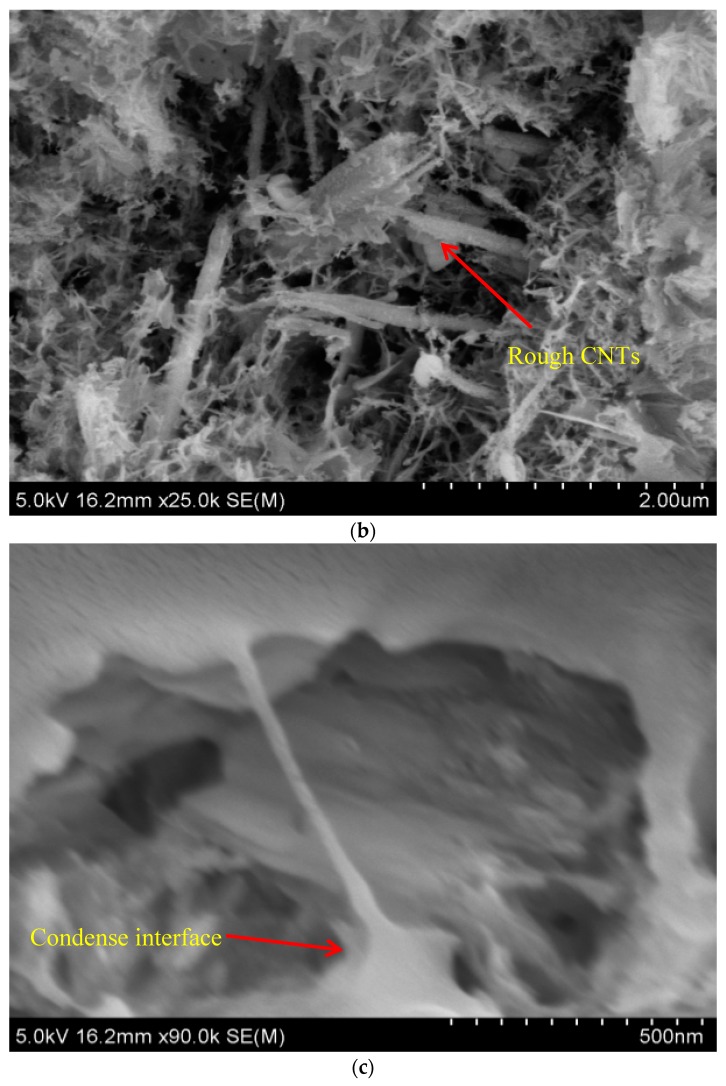

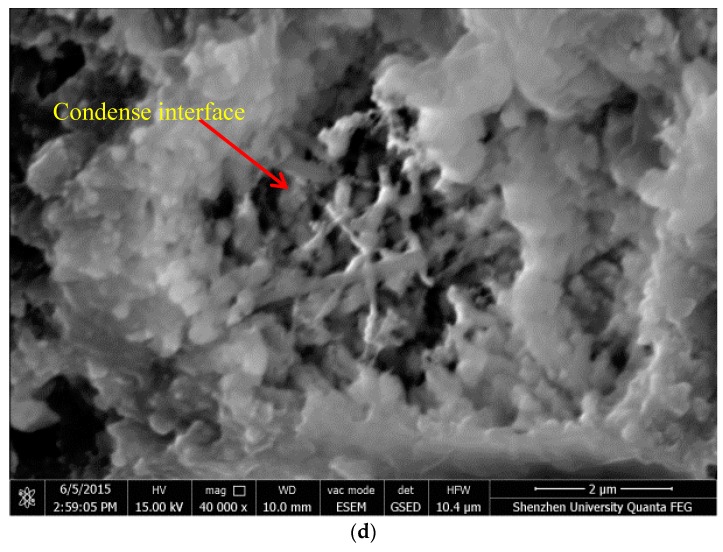
Scanning electron microscope (SEM) images of MWCNT/cement composites. (**a**) MWCNTs × 20 K; (**b**) MWCNTs + NS × 25 K; (**c**) MWCNTs + NS × 90 K; (**d**) MWCNTs + NS × 40 K.

Figure 3 presents the SEM micrographs of the MWCNT/cement composites with and without the assistance of NS for dispersion. From the micrographs presented, it can be observed that individual MWCNTs were firmly embedded in hydration products, which proved that MWCNTs were well-dispersed in the samples by means of the sonication method. In Figure 3a, individual MWCNTs in the hydrated cement paste with smooth surface can be observed. In comparison, as shown in Figure 3b, the individual MWCNTs dispersed by NS in the cement paste presented in a rough surface morphology. Figure 3c,d indicate that MWCNTs dispersed by NS in cement matrix had bridging and cross-linking effects, respectively.

It is noteworthy that the interface between MWCNTs and cement matrix was densified in Figure 3c,d when compared with the interface from our previous research [13]. As cracks go through the reinforced zone, the pullout performance of MWCNTs dissipates the energy working on crack development under loading. The direct nucleation on the MWCNTs bundles would produce a dense C–S–H structure against the surfaces that would be capable of improving the load-transfer capacity between MWCNTs and the cement matrix.

**Figure 4:**
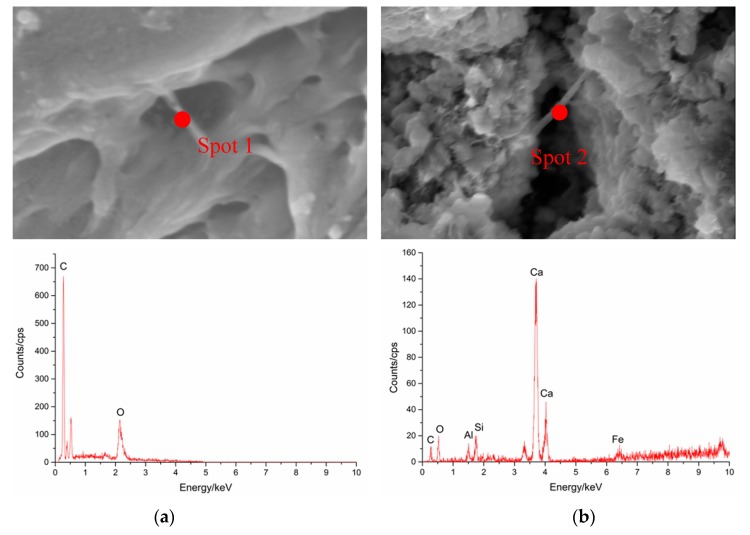
Energy dispersive spectrometer (EDS) results of MWCNT/cement composites without and with NS. (**a**) EDS spectrum (MW-W × 40 K); (**b**) EDS spectrum (MW-NG × 40 K).

The nano-fibers in the two samples present different morphologies. An energy dispersive spectrometer (EDS) was used to analyze the difference in the fibers, as shown in Figure 4. For pure MWCNTs with a smooth surface (Figure 4a), carbon was the most common element. In comparison, for the NS-conditioned sample that had the rough MWCNT surface (Figure 4b), elements included in hydration products can be detected, e.g., an obviously high content of Ca. The rough morphology observed in Figure 4b might be a nano-carbon fiber covered with hydration products of NS. NS is believed to adsorb on the surface of MWCNTs and improve density and C-S-H hydration through a pozzolanic reaction [27]. The NS acts as a media to improve the interaction between MWCNTs and C–S–H products, finally leading to a denser interface.

### 2.4. Damping Property

**Figure 5:**
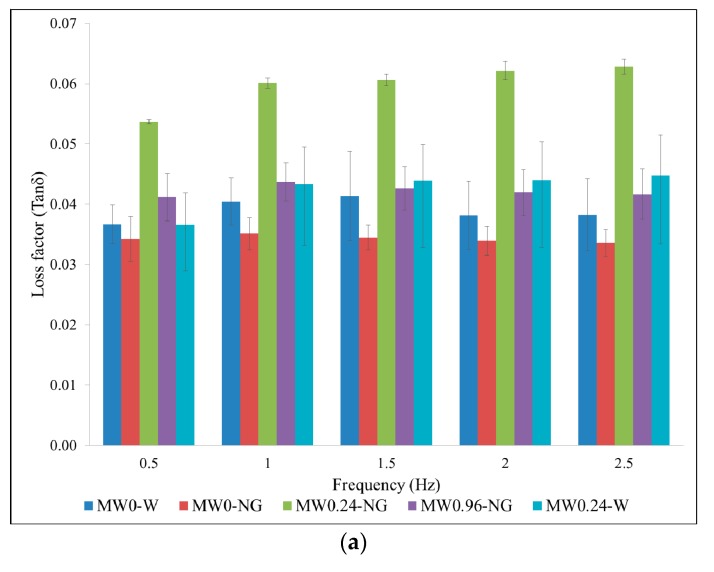

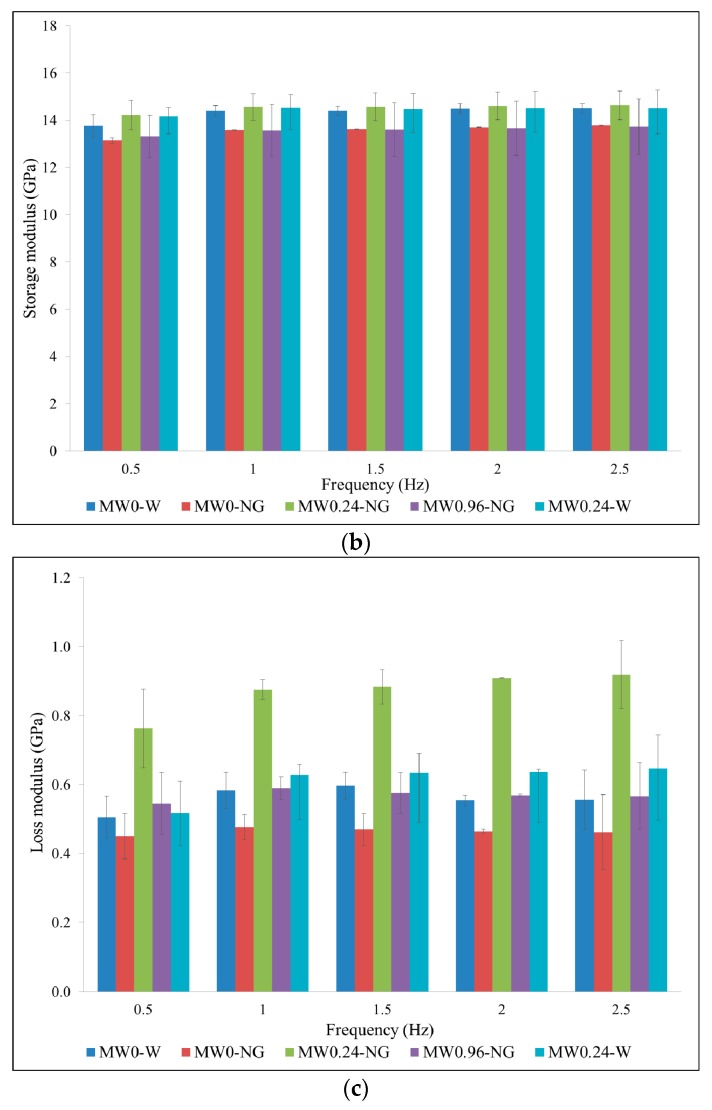
Damping property of MWCNT/cement composite. (**a**) Loss factor; (**b**) Storage modulus; (**c**) Loss modulus.

Figure 5 shows the results of the loss factor *Tan δ* (Figure 5a), the storage modulus *G* (Figure 5b) and the loss modulus *G″* (Figure 5c) of the cement composites, which were obtained from the DMA test. The results show that the vibration frequency applied to the samples during the DMA test had a limited influence on the results.

As seen from Figure 5a, the sample MW0.24-W had a higher loss factor than MW0-W. Based on Duan and Luo’s theory [31], this could be attributed to dislocations, multi-phase boundaries and multiform interfaces of the MWCNTs. The apparent internal friction forces among the MWCNT bundles and the external friction between MWCNTs and cement matrix might be another main reason. In Figure 5a, the sample MW0.24-NG exhibited a much higher loss factor than MW0.24-W, which indicates an improvement in the vibration-reduction property of MWCNT/cement composite due to the NS coating on MWCNTs. The sample MW0.96-NG has a relatively lower loss factor in comparison with the samples MW0.24-W and MW0.24-NG due to the agglomeration of MWCNTs, which was evidenced by the UV-Visible spectroscopy results in Section 2.1. The agglomerated MWCNTs will degrade the interaction with cement matrix and therefore reduce the friction force under an applied tension.

Figure 5b shows the variation of storage modulus at different frequencies. Comparing the MW0-W and MW0.24-W samples, the latter had a higher storage modulus, especially at low frequencies. This could mean that a relatively strong internal force was formed in MW0.24-W, which was due to be the interactive friction between MWCNTs and hydration products [32]. Meanwhile, it was found that MW0.24-NG presented a marginal higher value than MW0.24-W, indicating that incorporation of NS may increase the friction strength between MWCNTs and hydration products. In comparison, a relatively low storage modulus was observed in MW0.96-NG. The results indicated that the re-agglomeration of MWCNTs in high dosages will lead to poor connection between the MWCNTs and cement matrix, which did not exhibit any noticeable increase in material elasticity. 

From the results presented in Figure 5c, it can be observed that MWCNT/cement composites see a significant improvement in the loss modulus after adding NS. The enhancements in sample MW0.24-NG were 48% and 66% with respect to MW0.24-W and MW0-W, respectively. Therefore, addition of NS could improve the energy dissipation ability of MWCNT/cement composite.

## 3. Discussions

**Figure 6:**
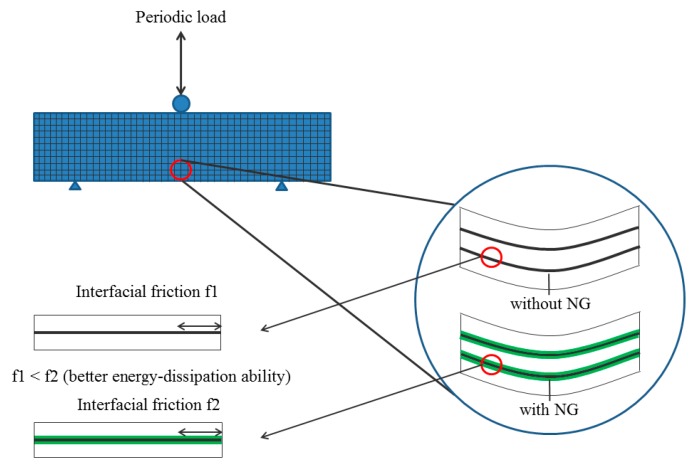
Schematic representation of damping property.

This study investigates the effects of NS on interfacial adhesion between MWCNTs and cement matrix using various methods. Prior to the tests, UV-Visible spectroscopy showed that addition of NS does not affect the dispersion of MWCNTs in suspension. A calorimetry test indicated that NS promotes the nucleation of C–S–H around MWCNTs in cement hydration. Hence, it is proposed in this study that NS may adsorb on and activate the surface of MWCNTs, which offered more active nuclei for formation of C–S–H on MWCNTs and condensed the interface between MWCNTs and cement matrix. Further investigations are carried out to prove this hypothesis. The morphology observation also proves that NS absorbs on the surface of MWCNTs and accelerates the formation of hydration products, leading to a rough MWCNT surface and compact interface between MWCNTs and cement matrix. Based on the previous findings [33], the damping mechanism in cement composite could be attributed to different sources of energy dissipation, including multiform interfaces of cement matrix, frictional damping due to slip-in the unbound regions and damping due to damaged cement matrix. For CNT-reinforced cement composite, addition of CNTs to the cement matrix would introduce diversified dislocation of cement matrix and additional friction damping in the interface. In this study, CNTs are dispersed in the same state and the samples are subjected to non-destructive periodic load, so both multiform interfaces and damping from damage would not contribute greatly to the difference of damping properties. During the small deformation, MWCNTs is able to form friction force with the cement matrix through marginal slippage and hence dissipate the vibration energy. Therefore, in this work, the frictional damping mechanism is highly expected to be the dominant factor affecting the damping properties of cement composite. Compared with the smooth surface of pure CNTs, the NS is able to densify the interface between CNTs and cement matrix through a pozzolanic reaction and enhance the capacity for load transfer [28]. The higher densification between MWCNTs and cement matrix produces better reinforcement of MWCNTs through higher interface frictional strength, which is responsible for the significant energy dissipation of cement composite [31], leading to higher loss modulus and better energy dissipation in DMA tests. Figure 6 shows the schematic representation of the damping properties tested in DMA test. The interface between the NG hydration product and MWCNTs was physically connected, which is supposed to be a new form of friction regime introduced into the composite and should contribute to greater energy dissipation during the DMA analysis. In conclusion, addition of NS can improve the interfacial adhesion between MWCNTs and cement matrix. Compared with the negative nucleation effects of silica-functional CNTs during the cement hydration [28], it is found that NS produces greater improvement in CNT nucleation, which may accelerate the early growth of cement composite strength. Due to the excellent damping ability, it is expected that NS-CNT-reinforced cement composite will be used for important civil infrastructure to alleviate the dynamic vibration response and damage or fatigue, in order to meet the safety and comfort needs of the people.

## 4. Materials and Methods

### 4.1. Materials 

The cement used in this study is Type I 42.5R Portland cement manufactured by Guangzhou Xinhe Co., Ltd. (Guangzhou, China). The carbon nanotubes are carboxylated multi-walled CNTs (MWCNTs) from Chengdu Organic Chemistry Research Institute (Chengdu, China). Their physical properties are provided by the manufacturer and are shown in Table 5. The nano-silica sol-gel (NS) is bought from Qingdao Yumin Ltd. (Qingdao, China) and its physical properties are provided by the manufacturer and presented in Table 6.

### 4.2. Methods

In this study, MWCNT/cement composites were prepared in two steps: (a) dispersion of MWCNTs in water for aqueous suspension; (b) manufacture of specimens by mixing cement with the obtained MWCNTs aqueous suspension.

#### 4.2.1. Preparation of Aqueous Dispersion

During the preparation, designated amount of MWCNTs was added into NS gel and water and the composite was mixed with a magnetic stirrer at a constant speed of 200 rpm/min for 10 min. The aqueous suspension of MWCNTs was successively sonicated with a probe sonicator of ultrasonic cell disrupter (JY92-IIN Ultrasonic cell disrupter, Ningbo Scientz Biotechnology Co., Ltd., Ningbo, China). For the sonication process, the total energy output was controlled at 125,000 J/mL and it was set in a cyclic stirring and standing regime, where the duration for each stage was 3 s.

#### 4.2.2. Manufacture of CNTs/Cement Composite

A water-to-cement ratio (*w*/*c*) of 0.4 was used for the specimens. One percent (1%) nano-silica by weight of cement was added, and the water content of NS suspension is considered in total water to cement ratio during casting. Plain cement pastes with NS were also prepared as controls. Manufacture and curing of samples followed the following procedures:

The obtained MWCNTs suspension was mixed with cement in a cement paste mixer (NJ-160A, Hebei Dahong Experimental Instrument Co., Ltd., Cangzhou, China) at a low speed of 140 ± 5 r/min for 1 min;

(a) Extra amount of water was added into the suspension and the composite was mixed at a high speed of 285 ± 10 r/min for 3 min;
(b) The fresh pastes were cast into molds with dimension of 100 × 12 × 5 mm for DMA test;
(c) The samples were demolded after 24 h and cured in a 20 °C and relative humidity of 95% environment until test.

### 4.3. Characterization of the Degree of MWCNT Dispersion

The degree of MWCNT dispersion in water or NS was evaluated based on the absorbance of characteristic peaks in the ultraviolet spectral region (at a length of 250 nm) [34,35] of a UV-Visible spectroscopy (LAMBADA 950, Perkin Elmer, Waltham, MA, USA). The tested substance of 1 mL was taken directly after sonication, and the substance was then diluted in 40 mL distilled water. 

Based on the authors’ knowledge, the MWCNTs aqueous suspension may be unstable when standing still in room environment and its properties will change along with standing duration. This change in degree of dispersion within the first 48 h was discussed in this study with a loss rate, *ΔA*, calculated based on following equation:

(1) $$\Delta A=\frac{A_{0}-A_{n}}{A_{0}}\times 100\%$$

where *A*_0_ and *A_n_* are the intensity for the absorbance of the characteristic peaks at a length of 250 nm of the samples tested immediately after the preparation and after standing in the room environment for *n* hours, respectively.

### 4.4. Calorimetry Test

2 g of water or aqueous suspension with different MWCNTs concentrations obtained after sonication was poured into a glass ampoule with 5 g of cement inside the calorimeter. The glass ampoule was, then, placed into the isothermal calorimeter (Toni 7338, Zwick Roell, Ulm, Germany). The instrument is set to a temperature of 25 ± 0.1 °C. After equilibrating whole system, the heat evolution of the cement pastes was tracked continuously for three days. Finally, the total rate of heat evolution during early-age cement hydration was recorded.

### 4.5. Morphology Observation

The scanning electron microscope (SEM) (SU-70, Hitachi, Tokyo, Japan) was used for study the influences of NS on morphology of MWCNTs in cement matrix. Also, energy dispersive spectrometer (EDS) was used to analyze the elemental composition of the fiber structure.

### 4.6. Dynamic Mechanical Analysis 

**Figure 7:**
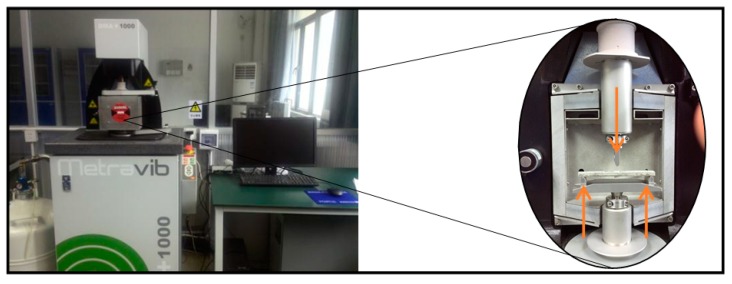
DMA instrument.

A dynamic mechanical analysis (DMA) instrument (DMA+1000, Metravib, Limonest, France), as shown in Figure 7, was used to determine the damping properties of materials on 100 × 12 × 5 mm samples. Damping refers to the loss of mechanical energy as the amplitude of motion gradually decreases. Samples were fixed in a three-point bending clamp and tested with a cyclic bending method, in which the temperature was set at 25 °C. The maximum deformation was 7 μm and the frequencies was at 0.5 Hz, 1.0 Hz, 1.5 Hz, 2.0 Hz and 2.5 Hz, respectively. During the analysis, the loss factor (*Tan δ*) and storage modulus (*G*) of MWCNT/cement composites were recorded directly by the machine. Then, a loss modulus, namely *G″*, a measure of the energy dissipated as heat in a viscoelastic material, can be calculated based on Equation (2).

(2) G″=Tanδ×G

where *Tan δ* is a measure of material damping, such as vibration or sound damping; *G* is a measure on elasticity, revealing ability of a sample in energy reservation; *G″* represents the capacity of a material to dissipate energy (mechanical or acoustic) as heat, owing to molecular motion within the sample that dissipate energy as heat. In this study, the *G″* is considered to be a comprehensive parameter to explore the effects of NS on MWCNT energy dissipation behavior in cement matrix.

## 5. Conclusions

In this study, damping properties were first investigated to study the influence of NS on the interactive behaviors between MWCNTs and cement matrix. Addition of NS to MWCNT-reinforced cement composite is proposed as an effective way to improve the interactive behaviors in the interface. The effect of NS on MWCNT dispersion in aqueous suspension was characterized using UV-Visible spectroscopy. Hydration heat and morphology were also investigated to demonstrate the nucleation effect and interface of MWCNTs. Damping properties were evaluated with a DMA test to demonstrate the energy dissipation capacity of MWCNT/cement composite. The following conclusions were drawn:

(a) For MWCNT dispersion in NS gel, the optimal dosage of MWCNTs in this study is 0.02% by weight of cement;
(b) Addition of NS does not affect the dispersion of MWCNTs in aqueous suspension;
(c) NS absorbs on the surface of MWCNTs and promotes the formation of hydration products on the surface of MWCNTs;
(d) NS improves the interfacial adhesion between MWCNTs and cement matrix, leading to higher loss modulus and improved energy dissipation ability. The property enhancements in the MW0.24-NG sample were 48% and 66% higher than in the MW0.24-W and MW0-W samples, respectively.

## Figures and Tables

**Table 1:** Proportions for aqueous dispersion of multi-walled carbon nanotubes (MWCNTs) (MW1 refers to 0.02% MWCNTs without nano-silica (NS); MW2 refers to 0.02% MWCNTs with NS; MW3 refers to 0.08% MWCNTs with NS).

Notation	MWCNTs (g)	Water (mL)	NG (mL)
MW1	0.24	100	0
MW2	0.24	0	100
MW3	0.96	0	100

**Table 2:** UV-Visible absorbance of multi-walled carbon nanotube (MWCNT) suspension during the initial 48 h standing.

Notation	0 h		3 h		12 h		24 h		48 h	
	*A*	*ΔA*	*A*	*ΔA*	*A*	*ΔA*	*A*	*ΔA*	*A*	*ΔA*
MW1-W	1.20	0	1.19	1%	1.18	2%	1.18	2%	1.17	3%
MW2-NG	1.33	0	1.34	−1% *	1.37	−4% *	1.36	−3% *	1.32	0
MW3-NG	1.20	0	0.32	73%	0.30	75%	0.29	76%	0.29	76%

* Negative *ΔA* values indicate increase in intensity of the UV-Visible absorbance.

**Table 3:** Mix proportions for CNTs/cement composite.

Notation	Cement (g)	MWCNTs (g)	Water (mL)	NG (mL)
MW0-W	1200	0	480	0
MW0-NG	1188	0	372	120
MW0.24-W	1200	0.24 (0.02%)	480	0
MW0.24-NG	1188	0.24 (0.02%)	372	120
MW0.96-NG	1188	0.96 (0.08%)	372	120

The weight ratio of MWCNTs to cement is calculated in the parenthesis.

**Table 4:** Summary of cumulative heat of hydration of MWCNT/cement composites.

Notation	MW0-W	MW0-NG	MW0.24-W	MW0.24-NG	MW0.96-NG
**Heat (J/g)**	296.97	292.4 (−1.54%)	313.13 (+5.44%)	314.36 (+5.86%)	304.28 (+2.46%)

**Table 5:** Properties of the MWCNTs.

Notation	Diameter	Length	Purity	Specific Surface Area	–COOH	Making Method
MWCNTs	10–20 nm	10–30 μm	>95%	>120 m^2^/g	2 wt %	CVD

**Table 6:** Properties of the NG.

Notation	SiO_2_/gel	Na_2_O	pH	Density	Average Diameter
NG	10%	0.18%	8.3	1.04 g/cm^3^	14 nm
